# Synthesis, characterization and supra­molecular analysis for (*E*)-3-(pyridin-4-yl)acrylic acid

**DOI:** 10.1107/S2056989024002627

**Published:** 2024-03-26

**Authors:** Valentina Florez-Muñoz, Andres Felipe Guerrero, Mario Macias, Luis Alberto Illicachi, Richard D’Vries

**Affiliations:** a Facultad de Ciencias Básicas, Universidad Santiago de Cali, Calle 5 No 62-00, Cali, Colombia; b Cristalografía y Química de Materiales (CrisQuimMat), Facultad de Ciencias, Departamento de Química, Universidad de los Andes, Cra. 1 No 18a-12, Bogotá, Colombia; c Facultad de Ciencias Naturales, Exactas y de la Educación, Departamento de Química, Universidad del Cauca, Calle 5 No 4-70, Popayán, Colombia; Universidade de Sâo Paulo, Brazil

**Keywords:** crystal structure, (*E*)-3-(pyridin-4-yl)acrylic acid, supra­molecular analysis

## Abstract

The title compound, C_8_H_7_NO_2_, crystallizes as prismatic colourless crystals in space group *P*  , with one mol­ecule in the asymmetric unit. The pyridine ring is fused to acrylic acid, forming an almost planar structure with an *E*-configuration about the double bond with a torsion angle of −6.1 (2)°. In the crystal, strong O—H⋯N inter­actions link the mol­ecules, forming chains along the [101] direction. Weak C—H⋯O inter­actions link adjacent chains along the [100] direction, generating an *R* ^2^_2_(14) homosynthon. Finally, π–π stacking inter­actions lead to the formation of the three-dimensional structure. The supra­molecular analysis was supported by Hirshfeld surface and two-dimensional fingerprint plot analysis, indicating that the most abundant contacts are associated with H⋯H, O⋯H/H⋯O, N⋯H/H⋯N and C⋯H/H⋯C inter­actions.

## Abstract 2

In the title compound, the pyridine ring is fused to acrylic acid, forming an almost planar structure with an *E*-configuration about the double bond. In the crystal, O—H⋯N and C—H⋯O inter­actions together with π–π stacking inter­actions lead to the formation of the three-dimensional structure.

## 1. Chemical context

Cinnamic acid and its derivatives have been used in several applications related to medicinal chemistry (Deng *et al.*, 2023 ▸), organic synthesis (Chen *et al.*, 2020 ▸), and coordination chemistry (Zhou *et al.*, 2016 ▸). Cinnamic acids are reactive mol­ecules due to possessing an unsaturated carbonyl moiety, which can be considered a Michael acceptor and benzene ring. Both make it possible to modify them, resulting in synthetic cinnamic acid derivatives with a broad range of biological properties, including anti­bacterial (Ruwizhi & Aderibigbe, 2020 ▸) anti­tuberculosis (Teixeira *et al.*, 2020 ▸), anti­malarial (Fonte *et al.*, 2023 ▸), anti­diabetic (Adisakwattana, 2017 ▸; Feng *et al.*, 2022 ▸), anti­cancer (Feng *et al.*, 2022 ▸), anti­fungal (Liu *et al.*, 2024 ▸), Alzheimer’s treatment (Drakontaeidi & Pontiki, 2024 ▸), anti­oxidant (Nouni *et al.*, 2023 ▸), and cosmetic (Gunia-Krzyżak *et al.*, 2018 ▸). Among the various types of cinnamic acids documented, 4-pyridyl­acrylic acid (4-Hpya) is considered a highly valuable ligand because of several structural characteristics that make it suitable for the construction of coord­ination compounds. These characteristics include multiple coordination sites, which enable the formation of higher-dimensional structures, and versatile coordination modes to form different structures (Khalfaoui *et al.*, 2021 ▸). On the other hand, its capacity to function as both a hydrogen-bond donor and acceptor facilitates the creation of intricate hydrogen-bonded networks (Jiao *et al.*, 2007 ▸; Zhu *et al.*, 2005 ▸). 
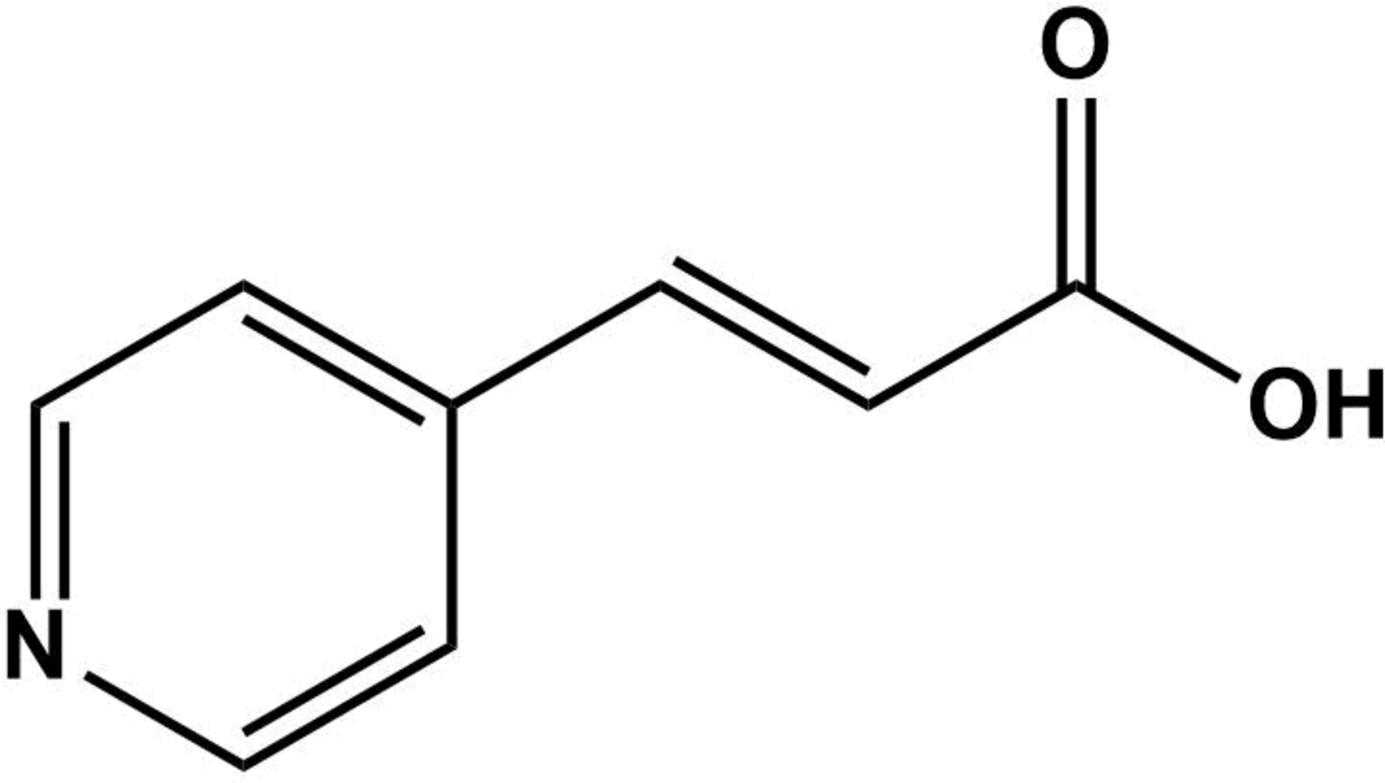


## 2. Structural commentary

**Figure 1:**
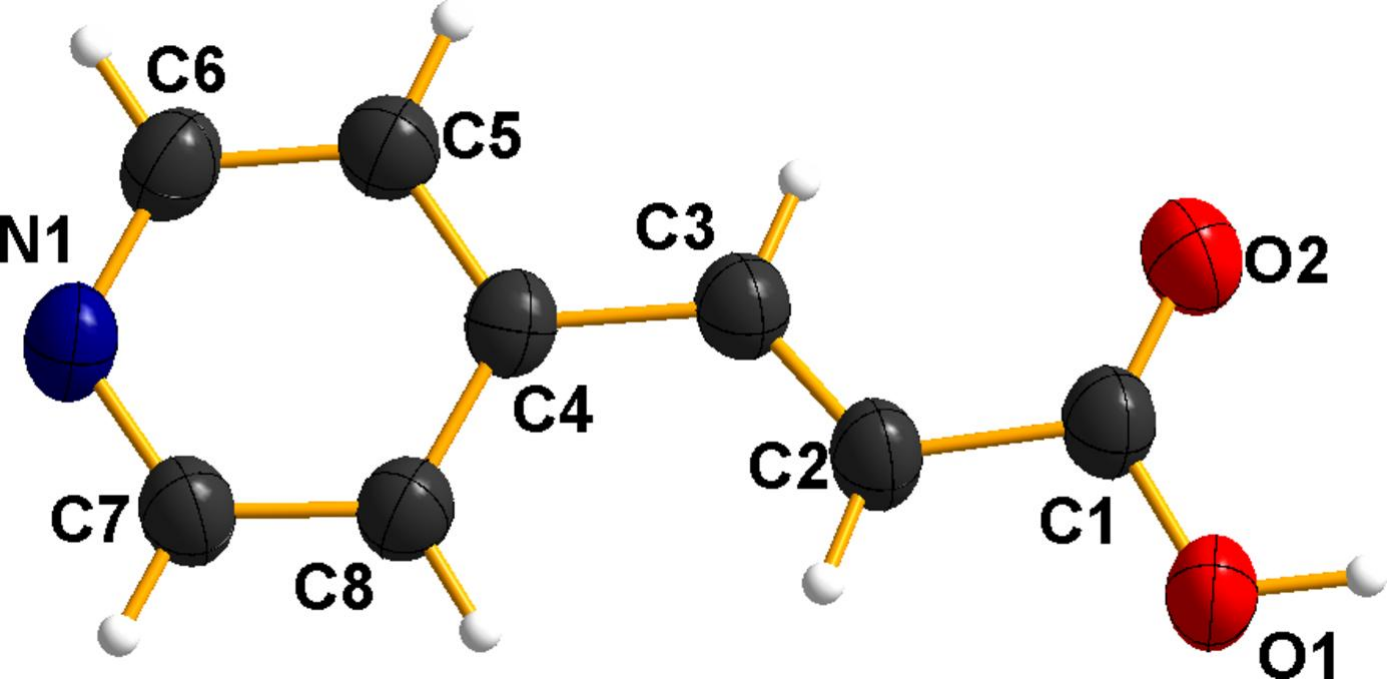
The mol­ecule of (*E*)-3-(pyridin-4-yl)acrylic acid compound with displacement ellipsoids drawn at the 50% probability level.

The title compound crystallizes in space group *P* 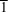 with one mol­ecule per asymmetric unit (Fig. 1 ▸). The pyridinic ring is fused to acrylic acid, forming an almost planar structure with an *E*-configuration about the double bond, with a C8—C4—C3—C2 torsion angle of −6.1 (2)°.

## 3. Supra­molecular features

**Figure 2:**
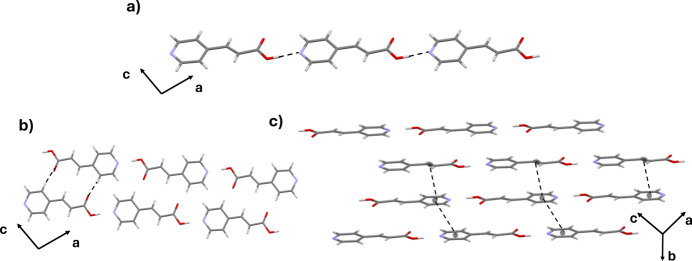
Supra­molecular inter­actions in the title compound. (*a*) O—H⋯N inter­actions forming chains, (*b*) two chains joined by C—H⋯O inter­actions and (*c*) π–π stacking inter­actions between the pyridine rings and the acrylic group.

In the crystal, strong O1—H1⋯N1 inter­actions link the mol­ecules, forming chains along the [101] direction (Fig. 2 ▸ *a*, Table 1 ▸). Adjacent chains are linked along the [100] direction through weak C—H⋯O inter­actions, generating an 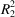 (14) homosynthon (Fig. 2 ▸ *b*). Finally, the three-dimensional supra­molecular structure is finally formed by slipped π–π stacking inter­actions (Hunter & Sanders, 1990 ▸) between the pyridinic rings (N1/C4–C8) with distances of 3.8246 (10) Å, and π–π stacking inter­actions of the acrylic double bond (C2=C3) of 3.4322 (10)Å (Fig. 2 ▸ *c*). An inter­action between the nitro­gen atom of the pyridinic ring, N1, and the double bond of the acrylic group with a distance of 3.4044 (13) Å is also observed.

**Figure 3:**
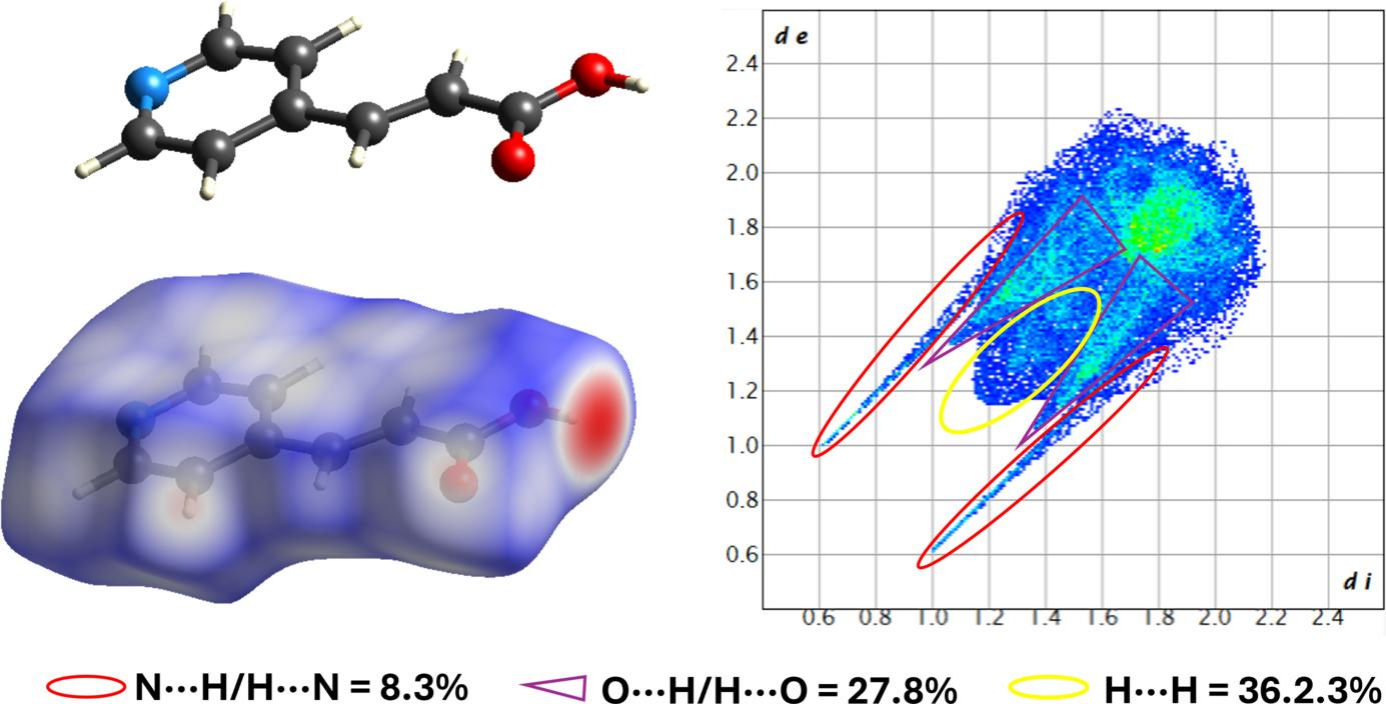
Hirshfeld surface and fingerprint plot analysis for the title compound.

A Hirshfeld surface analysis was performed to confirm, visualise and qu­antify the supra­molecular inter­actions present in title compound. The Hirshfeld surface mapped over *d*_norm_ and 2D fingerprint plots (Spackman & Jayatilaka, 2009 ▸) were generated using *Crystal Explorer 17* (Spackman, *et al.*, 2021 ▸). Fig. 3 ▸ shows the strongest inter­actions as red spots. These are associated with the donor and acceptor atoms, in this case for the O—H⋯N inter­action. The weakest inter­actions, associated with the C—H⋯O contacts, are shown as white areas. These inter­actions were qu­anti­fied through the fingerprint plots, indicating that the most abundant contacts are associated with H⋯H inter­actions (36.2%) while O⋯H/H⋯O, N⋯H/H⋯N and C⋯H/H⋯C inter­actions represent 27.8%, 8.7% and 10.7%, respectively. These results show that crystal packing is governed mainly by dispersion and electrostatic inter­actions.

## 4. Database survey

A search of the Cambridge Structural Database (Version 2023.3.0; Groom *et al.*, 2016 ▸) using Conquest (Bruno *et al.*, 2002 ▸) found seven entries for (*E*)-3-(pyridin-4-yl)acrylic acid derivative mol­ecules. In all cases, the protonation of the nitro­gen atom in the pyridine ring leads to the formation of pyridinium salts. These include halides (Hu, 2010 ▸; Kole *et al.*, 2010 ▸), tri­fluoro­acetate (Kole *et al.*, 2010 ▸), hydrogen sulfate (Kole *et al.*, 2010 ▸), perchlorate and hexa­fluoro­phosphate (Kole *et al.*, 2011 ▸).

## 5. Synthesis and crystallization

**Figure 4:**
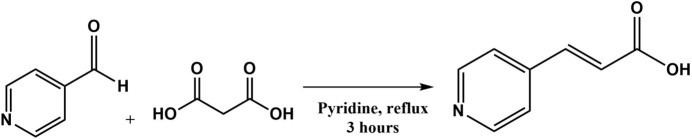
Reaction scheme for obtaining (*E*)-3-(pyridin-4-yl)acrylic acid.

The synthesis of (*E*)-3-(pyridin-4-yl)acrylic acid compound was performed following the procedure reported by Kudelko *et al.* (2015 ▸) for the synthesis of 3-(pyrid­yl)acrylic acids (Fig. 4 ▸). In a 25 mL flat-bottomed flask, 728 mg of malonic acid (0.335 mmol) and 300 mg of 4-pyridincarb­oxy­aldehyde (0.33 5 mmol) were mixed with 2 ml of pyridine. The reaction mixture was refluxed under constant stirring for 3 h. The reaction synthesis was ice-cooled, and then drops of 37% HCl were added until precipitate formation was observed. The obtained solid was separated by filtration and washed with acetone. The solid product was recrystallized by slow water evaporation, giving a colourless crystalline powder and small prismatic crystals in 97.9% yield.

## 6. Refinement

Crystal data, data collection and structure refinement details are summarized in Table 2 ▸. The O-bound hydrogen atom (H1) was found in electron density maps and freely refined. C-bound hydrogen atoms were positioned geometrically and refined using a riding model [C—H = 0.93 Å, *U*_iso_(H) = 1.2*U*_eq_(C)].

## Supplementary Material

Crystal structure: contains datablock(s) I. DOI: 10.1107/S2056989024002627/ex2082sup1.cif

Structure factors: contains datablock(s) I. DOI: 10.1107/S2056989024002627/ex2082Isup2.hkl

Supporting information file. DOI: 10.1107/S2056989024002627/ex2082Isup3.cml

CCDC reference: 2341592

Additional supporting information:  crystallographic information; 3D view; checkCIF report

## Figures and Tables

**Table 1:** Hydrogen-bond geometry (Å, °)

*D*—H⋯*A*	*D*—H	H⋯*A*	*D*⋯*A*	*D*—H⋯*A*
O1—H1⋯N1^i^	0.99 (3)	1.63 (3)	2.6147 (18)	177 (3)
C5—H5⋯O2^ii^	0.93	2.57	3.336 (2)	140

Symmetry codes: (i) 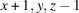 ; (ii) 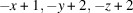 .

**Table 2:** Experimental details

Crystal data	
Chemical formula	C_8_H_7_NO_2_
*M*_r_	149.15
Crystal system, space group	Triclinic, *P* 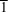
Temperature (K)	291
*a*, *b*, *c* (Å)	6.6279 (15), 7.3272 (12), 8.2308 (15)
α, β, γ (°)	67.271 (17), 83.403 (17), 73.006 (17)
*V* (Å^3^)	352.57 (13)
*Z*	2
Radiation type	Cu *K*α
μ (mm^−1^)	0.85
Crystal size (mm)	0.09 × 0.06 × 0.05

Data collection	
Diffractometer	SuperNova, Dual, Cu at home/near, Atlas
Absorption correction	Multi-scan (*CrysAlis PRO*; Rigaku OD, 2021 ▸)
*T*_min_, *T*_max_	0.831, 1.000
No. of measured, independent and observed [*I* > 2σ(*I*)] reflections	3635, 1461, 1243
*R*_int_	0.035
(sin θ/λ)_max_ (Å^−1^)	0.631

Refinement	
*R*[*F* ^2^ > 2σ(*F* ^2^)], *wR*(*F* ^2^), *S*	0.049, 0.153, 1.04
No. of reflections	1461
No. of parameters	104
H-atom treatment	H atoms treated by a mixture of independent and constrained refinement
Δρ_max_, Δρ_min_ (e Å^−3^)	0.14, −0.27

Computer programs: *CrysAlis PRO* (Rigaku OD, 2021 ▸), *SHELXT* (Sheldrick, 2015*a* ▸), *SHELXL2016/6* (Sheldrick, 2015*b* ▸) and *OLEX2* (Dolomanov *et al.*, 2009 ▸).

## supplementary crystallographic information

### Crystal data

**Table:**

C_8_H_7_NO_2_	*F*(000) = 156
*M**_r_* = 149.15	*D*_x_ = 1.405 Mg m^−^^3^
Triclinic, *P*1	Melting point: 553 K
*a* = 6.6279 (15) Å	Cu *K*α radiation, λ = 1.54184 Å
*b* = 7.3272 (12) Å	Cell parameters from 1657 reflections
*c* = 8.2308 (15) Å	θ = 6.8–74.9°
α = 67.271 (17)°	µ = 0.85 mm^−^^1^
β = 83.403 (17)°	*T* = 291 K
γ = 73.006 (17)°	Prismatic, colourless
*V* = 352.57 (13) Å^3^	0.09 × 0.06 × 0.05 mm
*Z* = 2	

### Data collection

**Table:**

SuperNova, Dual, Cu at home/near, Atlas diffractometer	1461 independent reflections
Radiation source: micro-focus sealed X-ray tube, SuperNova (Cu) X-ray Source	1243 reflections with *I* > 2σ(*I*)
Mirror monochromator	*R*_int_ = 0.035
Detector resolution: 10.6144 pixels mm^-1^	θ_max_ = 76.6°, θ_min_ = 5.8°
ω scans	*h* = −8→7
Absorption correction: multi-scan (CrysAlisPro; Rigaku OD, 2021)	*k* = −8→9
*T*_min_ = 0.831, *T*_max_ = 1.000	*l* = −10→10
3635 measured reflections	

### Refinement

**Table:**

Refinement on *F*^2^	0 restraints
Least-squares matrix: full	Hydrogen site location: mixed
*R*[*F*^2^ > 2σ(*F*^2^)] = 0.049	H atoms treated by a mixture of independent and constrained refinement
*wR*(*F*^2^) = 0.153	*w* = 1/[σ^2^(*F*_o_^2^) + (0.0977*P*)^2^ + 0.0215*P*] where *P* = (*F*_o_^2^ + 2*F*_c_^2^)/3
*S* = 1.04	(Δ/σ)_max_ < 0.001
1461 reflections	Δρ_max_ = 0.14 e Å^−^^3^
104 parameters	Δρ_min_ = −0.27 e Å^−^^3^

### Special details

**Table:**

Geometry. All esds (except the esd in the dihedral angle between two l.s. planes) are estimated using the full covariance matrix. The cell esds are taken into account individually in the estimation of esds in distances, angles and torsion angles; correlations between esds in cell parameters are only used when they are defined by crystal symmetry. An approximate (isotropic) treatment of cell esds is used for estimating esds involving l.s. planes.

### Fractional atomic coordinates and isotropic or equivalent isotropic displacement parameters (Å^2^)

**Table:**

	*x*	*y*	*z*	*U*_iso_*/*U*_eq_	
O1	0.48195 (18)	0.6974 (2)	0.64537 (15)	0.0609 (4)	
O2	0.63738 (19)	0.8136 (2)	0.79722 (16)	0.0669 (4)	
C2	0.3116 (2)	0.7317 (2)	0.89960 (18)	0.0484 (4)	
H2	0.211812	0.676180	0.879516	0.058*	
N1	−0.20475 (19)	0.72846 (17)	1.42131 (15)	0.0504 (4)	
C5	0.1186 (2)	0.8159 (2)	1.31387 (18)	0.0490 (4)	
H5	0.228403	0.862808	1.329376	0.059*	
C4	0.1149 (2)	0.76641 (18)	1.16689 (16)	0.0426 (3)	
C7	−0.2089 (2)	0.6805 (2)	1.28079 (19)	0.0507 (4)	
H7	−0.320055	0.632669	1.269716	0.061*	
C1	0.4937 (2)	0.75386 (19)	0.77647 (17)	0.0477 (4)	
C3	0.2868 (2)	0.7884 (2)	1.03649 (18)	0.0456 (3)	
H3	0.386535	0.847081	1.051782	0.055*	
C8	−0.0552 (2)	0.6991 (2)	1.15114 (18)	0.0483 (4)	
H8	−0.065343	0.666782	1.054192	0.058*	
C6	−0.0433 (3)	0.7943 (2)	1.43650 (18)	0.0524 (4)	
H6	−0.038863	0.827444	1.534078	0.063*	
H1	0.600 (4)	0.713 (4)	0.561 (3)	0.099 (8)*	

### Atomic displacement parameters (Å^2^)

**Table:**

	*U*^11^	*U*^22^	*U*^33^	*U*^12^	*U*^13^	*U*^23^
O1	0.0601 (7)	0.0851 (8)	0.0546 (6)	−0.0366 (6)	0.0209 (5)	−0.0374 (5)
O2	0.0631 (7)	0.0930 (9)	0.0644 (7)	−0.0440 (6)	0.0193 (5)	−0.0389 (6)
C2	0.0493 (8)	0.0521 (7)	0.0490 (8)	−0.0225 (6)	0.0125 (6)	−0.0213 (6)
N1	0.0545 (7)	0.0513 (6)	0.0463 (6)	−0.0184 (5)	0.0129 (5)	−0.0197 (5)
C5	0.0523 (8)	0.0528 (7)	0.0489 (7)	−0.0218 (6)	0.0056 (6)	−0.0225 (6)
C4	0.0457 (7)	0.0396 (6)	0.0405 (7)	−0.0122 (5)	0.0055 (5)	−0.0139 (5)
C7	0.0482 (7)	0.0572 (7)	0.0524 (7)	−0.0224 (6)	0.0101 (6)	−0.0233 (6)
C1	0.0508 (7)	0.0488 (7)	0.0451 (7)	−0.0201 (5)	0.0097 (5)	−0.0167 (5)
C3	0.0455 (7)	0.0468 (6)	0.0450 (7)	−0.0168 (5)	0.0058 (5)	−0.0161 (5)
C8	0.0513 (7)	0.0552 (7)	0.0453 (7)	−0.0202 (6)	0.0075 (5)	−0.0240 (5)
C6	0.0621 (9)	0.0553 (7)	0.0458 (7)	−0.0199 (6)	0.0091 (6)	−0.0249 (6)

### Geometric parameters (Å, º)

**Table:**

O1—C1	1.3135 (17)	C5—C4	1.3954 (18)
O1—H1	0.98 (3)	C5—C6	1.386 (2)
O2—C1	1.2110 (19)	C4—C3	1.4729 (19)
C2—H2	0.9300	C4—C8	1.392 (2)
C2—C1	1.4887 (18)	C7—H7	0.9300
C2—C3	1.322 (2)	C7—C8	1.384 (2)
N1—C7	1.3374 (18)	C3—H3	0.9300
N1—C6	1.332 (2)	C8—H8	0.9300
C5—H5	0.9300	C6—H6	0.9300
			
C1—O1—H1	113.5 (14)	C8—C7—H7	118.6
C1—C2—H2	118.9	O1—C1—C2	112.14 (13)
C3—C2—H2	118.9	O2—C1—O1	124.20 (13)
C3—C2—C1	122.27 (14)	O2—C1—C2	123.65 (13)
C6—N1—C7	117.87 (12)	C2—C3—C4	125.60 (14)
C4—C5—H5	120.4	C2—C3—H3	117.2
C6—C5—H5	120.4	C4—C3—H3	117.2
C6—C5—C4	119.18 (13)	C4—C8—H8	120.2
C5—C4—C3	119.41 (13)	C7—C8—C4	119.52 (12)
C8—C4—C5	117.36 (12)	C7—C8—H8	120.2
C8—C4—C3	123.22 (12)	N1—C6—C5	123.17 (12)
N1—C7—H7	118.6	N1—C6—H6	118.4
N1—C7—C8	122.88 (14)	C5—C6—H6	118.4
			
N1—C7—C8—C4	1.3 (2)	C3—C2—C1—O2	4.3 (2)
C5—C4—C3—C2	174.27 (12)	C3—C4—C8—C7	179.26 (12)
C5—C4—C8—C7	−1.2 (2)	C8—C4—C3—C2	−6.1 (2)
C4—C5—C6—N1	−0.2 (2)	C6—N1—C7—C8	−0.8 (2)
C7—N1—C6—C5	0.3 (2)	C6—C5—C4—C3	−179.79 (12)
C1—C2—C3—C4	−178.21 (11)	C6—C5—C4—C8	0.6 (2)
C3—C2—C1—O1	−176.96 (13)		

### Hydrogen-bond geometry (Å, º)

**Table:**

*D*—H···*A*	*D*—H	H···*A*	*D*···*A*	*D*—H···*A*
O1—H1···N1^i^	0.99 (3)	1.63 (3)	2.6147 (18)	177 (3)
C5—H5···O2^ii^	0.93	2.57	3.336 (2)	140

Symmetry codes: (i) *x*+1, *y*, *z*−1; (ii) −*x*+1, −*y*+2, −*z*+2.

### Hydrogen bonds and interactions geometry, (Å, °)

**Table:**

D—H···A	D—H	H···A	D···A	D—H···A
O1—H1···N1i	0.990 (3)	1.630 (3)	2.61470 (18)	177.0 (3)
C5—H5···O2ii	0.9300	2.5700	3.3360 (2)	140.00
Cp1···Cp1			3.82460 (10)	
Cp2···Cp2			3.43220 (10)	
N1···Cp1			3.40440 (13)	
